# Effect of Household Air Pollution and Neighbourhood Deprivation on the Risk of Acute Respiratory Infection Among Under-Five Children in Chad: A Multilevel Analysis

**DOI:** 10.3390/ijerph22050710

**Published:** 2025-05-01

**Authors:** Olatunde Aremu, Omolara O. Aremu

**Affiliations:** 1 Department of Public Health, Faculty of Health Education and Life Sciences, Birmingham City University, Birmingham B15 3TN, UK; 2 Zoonoses, Health & Infectious Disease Control, City Operations, Birmingham City Council, Birmingham B1 1BB, UK; omolara.aremu@birmingham.gov.uk

**Keywords:** children, household air pollution, acute respiratory infection, neighborhood disadvantage, polluting fuel, Chad

## Abstract

Background: Exposure to household air pollution (HAP) is one of the primary risk factors for acute lower respiratory infection (ARI) morbidity and mortality among children in low-income settings. This study aimed to examine the relative contribution of residing in deprived neighbourhoods and exposure to HAP on the occurrence of ARI among children using data from the 2014–2015 Chad Demographic and Health Survey (DHS). Methods: We applied multilevel modelling techniques to survey data of 2882 children from 372 communities to compute the odds ratio (OR) for the occurrence of ARI between children of respondents exposed to clean fuels (e.g., electricity, liquid petroleum gas, natural gas, and biogas) and respondents exposed to polluting fuel (e.g., kerosene, coal/lignite, charcoal, wood, straw/shrubs/grass, and animal dung). Results: The results showed that children exposed to household polluting fuels in Chad were 215% more likely to develop ARI than those not exposed to household air pollution (OR = 3.15; 95% CI 2.41 to 4.13). Further analysis revealed that the odds of ARI were 185% higher (OR = 2.85; 95% CI 1.73 to 4.75) among children living in rural residents and those born to teenage mothers (OR = 2.75; 95% CI 1.48 to 5.15) who were exposed to household polluting fuels compared to their counterparts who were not exposed. In summary, the results of the study show that the risk of ARI is more common among children who live in homes where household air-polluting cooking fuel is widely used, those living in rural areas, those living in socioeconomically deprived neighbourhoods and from the least wealthy households, and those born to teenage mothers in Chad. Conclusions: In this study, an independent relative contribution of variables, such as HAP from cooking fuel, neighbourhood deprivation, living in rural areas, being from a low-income household, having a mother who is a manual labourer worker, and being given birth to by a teenage mother, to the risk of ARI among children is established.

## 1. Introduction

Although childhood morbidity and mortality are global problems, the difference in childhood deaths between the less developed and economically endowed regions is evident [1] of the estimated half a million under-five deaths that occur worldwide, where nearly all are in low- and middle-income countries (LMICS) [1,2,3]. Most of these deaths and the disease burden among children are mainly due to modifiable factors. Children’s health outcomes, including their chances of survival, depend primarily on the nature of the household environment in which they reside [1,4]. Several factors, such as the availability of potable drinking water, secured toilet facilities, and non-polluting cooking fuel, can shape the household environment. Sadly, these amenities are lacking in most LMICS [5,6]. Even when present, they are sources of either household injuries or health hazards. As a result, a more significant number of these children die before reaching their fifth birthday from environmental-induced health problems, such as diarrhoea, malaria, and acute respiratory infections (ARIs) [5,7,8].

Together, these three infectious diseases contribute significantly to the global burden of the disease [3,9,10,11]. Deaths caused by these diseases are largely preventable through breastfeeding practices, vaccinations, hand washing with soap, safe drinking water, and basic sanitation [12,13]. A clean home environment and adequate ventilation are crucial to reducing the transmission of pathogens that cause diarrhoea, ARI, and fever [14].

More than ever before, solid fuel use has increased considerably worldwide [15]. According to estimates, at least three billion people worldwide rely on solid fuels, such as wood, animal dung, crop residues, charcoal, and coal, for cooking and heating [15,16]. Nearly three-quarters of users reside mainly in the developing regions of sub-Saharan Africa and Asia [16]. Unlike clean fuels, biomass fuels rank low on the energy ladder regarding combustion efficiency and cleanliness [17]. Many studies on household air pollution (HAP) link the incomplete combustion of biomass fuel to undesirable health outcomes [5,18]. HAP from cooking fuel is one of the potential risk factors that increase the severity of ARI by destroying respiratory tract defences and making children, especially those under the age of five, more susceptible to disease-causing microorganisms [19]. In sub-Saharan Africa (SSA), as well as other resource-limited settings, women and young children are disproportionately disadvantaged in terms of their vulnerability to the damaging effects of HAP [10]. Although evidence has shown that continuous exposure to HAP may cause adverse health effects and impact the health of women and their unborn children, these adverse effects include increasing the severity of ARI, especially among children under the age of five [5,18,19]. Children under five spend more time indoors at home and are more vulnerable to several health problems, such as recurrent respiratory infections, due to indoor pollution [20].ARIs represent a significant economic burden to households, and the financial hardship caused by ARIs is untold [3].

Chad is a landlocked country in Central Africa. As with several other countries in the region, more than 85% of the population depends on solid fuels for cooking and their energy needs [21]. According to estimates from the Global Burden of Disease (GBoD) study, the PM_2.5_ (that is, particulate matter smaller than 2.5 microns in diameter), a measure of mean annual air pollution exposure for Chad, was 64.1 (micrograms per cubic meter) [22]. This level of pollution places Chad among the countries with the worst air quality compared to others worldwide [22,23]. Chad has several policies and programmes to control air pollution and improve air quality [21,24]. These policies have focused on reducing the emissions from industries, transportation, and open waste burning. However, HAP continues to be responsible for an estimated 9600 premature deaths every year in the country [22]. As a result, coupled with other factors, Chad could not meet the Millennium Development Goal of reducing under-five mortalities [5,25]. Several studies have been conducted, but none of these have explored the effect of neighbourhood socioeconomic disadvantage, a marker of deprivation, and exposure to HAP due to cooking fuel on the risk of ARIs. A thorough understanding of the influence of deprivation on the occurrence of childhood infectious diseases such as ARIs is essential. Currently, there is no information to inform policy on factors associated with exposure to HAP in Chad. Reducing household air pollution and its adverse effects on under-five mortality are key targets in Sustainable Development Goal 3. Therefore, this study aims to provide evidence and examine available data to understand the association between HAP, individual, household and neighbourhood SEP, various household-level factors, and the risk of ARI among children under five in Chad. Insight into such information will be of policy relevance to policymakers and environmental health planners in Chad and other resource-limited settings.

## 2. Materials and Methods

### 2.1. Study Design and Sampling Technique

This study was based on the 2014–2015 Chad Republic Demographic and Health Survey (CDHS). The sample size for this research was limited to individual data on 2882 mothers aged 15–45 and 2882 children aged 0–59 months from 372 communities. Demographic and Health Surveys, commonly referred to as DHS, are a series of nationally representative surveys typically conducted in most low- and middle-income countries by various in-country bodies with technical assistance from ICF Macro International. In the case of Chad, since 1993, the authority of the Institut National de la Statistique, des Études Économiques et Démographiques (INSEED) in N’Djamena, with technical assistance from ICF International in Maryland, USA, has been conducting Demographic and Health Surveys [26]. The United States Agency for International Development (USAID) provided financial assistance, and the Global Fund funded the field surveys reported in this study. Briefly, the CDHS was conducted using a multistage stratified sampling procedure to select a randomly stratified sample of clusters. In the initial stage, a probability proportional to the size (number of households within each cluster) was used to select the cluster. Clusters are administratively defined areas sometimes used to denote communities or neighbourhoods. The second phase systematically sampled households in urban and rural areas from each cluster. Following this, a face-to-face personal interview was conducted with a random sample of women aged 15–49 years and men aged 15–59 years in households from each cluster, using a semi-structured questionnaire. The questionnaire, which had been translated into major local languages, detailed various questions relating to the respondents′ socio-demographic and health characteristics. All participants were informed of the purpose of the survey and allowed to give their consent before the interview commenced. The full details of the methodology and procedures used for sample collection in the CDHS have been published elsewhere [26].

### 2.2. Outcome Variable

The study’s primary outcome variable was the probability of a child developing respiratory symptoms, such as rapid breathing, blocked nose, runny nose, and catarrh, two weeks prior to the interview date. The responses were coded as “1” = Yes or “0” = No.

### 2.3. Exposure Variable

The primary exposure variable was the cooking fuel used in households. As recorded in the DHS survey questionnaire, the respondents were asked, “What type of fuel does your household mainly use for cooking?” In response, 12 types of cooking fuel have been reported. In this study, cooking fuels were grouped into two categories: “clean fuels” (electricity, liquid petroleum gas, natural gas, and biogas) and “polluting fuels” (kerosene, coal/lignite, charcoal, wood, straw/shrubs/grass, crops, and animal dung). Previous studies have reported kerosene as a polluting fuel and found significant associations between HAP and ARIs in children and kerosene fuel use [7]. Therefore, kerosene is categorised as a polluting fuel.

### 2.4. Covariates

The DHS did not collect direct information on household income and expenditure. Consequently, the DHS wealth index was used as a proxy indicator for the socioeconomic position of the participants, the mother’s education (categorised as “secondary or higher”, “primary”, or “no education”) and the mother’s working status (“manual”, not working”, professional”) were included as markers of socioeconomic status. Place of residence (categorised as “urban” or “rural”), and “breastfeeding status”(categorised as ever, never, and still breastfeeding “yes” or “no”). Other variables include “parity (categorised as 1–3 and 4+), “place of delivery” (categorised as home or hospital), “maternal access to media” (categorised as none, 1, 2, or 3), “child’s gender” (categorised as male or female), maternal age (years) (categorised as 35+, 25–34, 15–24), and “child’s age (months)” categorised as 0–10, 11–21, 22–32, and 33+.

The neighbourhood socioeconomic disadvantage index was developed using principal component analysis (PCA) [27]. The index comprises four variables: the proportion of respondents living in rural areas, the proportion of respondents who were not working, the proportion of respondents living below the poverty level (those below the 20% quintile on the wealth index), and the proportion of respondents with no education. Several scholars have widely adopted this index to examine the effect of neighbourhood socioeconomic status on health [28,29,30]. The standard scores generated from the continuous index had a mean value of 0 and a standard deviation of 1. These were then used to stratify neighbourhoods into two categories: most socioeconomically disadvantaged neighbourhoods, if the scores were higher than 1, and least socioeconomically disadvantaged neighbourhoods, if the scores on the index were lower than 0.

### 2.5. Statistical Analysis/Analytical Procedure

Multilevel logistic regression modelling and model specification

Given the hierarchical structure of the sample and the binary outcome, a multilevel logistic modelling approach was adopted. A two-level model written below was specified as follows:

(1) $$\log it\;\left[\pi ijk\right]=log[\frac{\pi ijk}{1-\pi ijk\;}]=\beta 0+Xijk+u0jk+v0k$$

where ***π_ijk_*** is the probability of child *i* of the mother residing in household *j* in community k developing symptoms of ARI. The right-hand side of the equation comprises the fixed parts ***X****_ijk_* and ***β***_0._ ***X****_ijk_* is a vector of covariates corresponding to child *i* of the mother residing in household *j* in community k, while ***β***_0_ is a vector of unknown parameter, ***u***_0jk_ is the random effect at the mother level, and ***v***_0k_ is the random effect at the neighbourhood level. The intercept, or the average probability of a child developing ARI symptoms, varies randomly between children in each household and between households within neighbourhoods. This analysis was performed in three steps. In Model 0 (the base model), no explanatory variables were included; it focused solely on decomposing the total variance into its component parts, namely the child and neighbourhood components.

In Model 1, only children and mothers’ and household-level variables were included. In Model 2, neighbourhood-level variables were added to Model 1. The measures of association are shown as odds ratios (ORs) with 95% confidence intervals (CIs). The results of the measures of variation are presented as the variance partition coefficient (VPC) and percentage change in variance (PCV). All parameters were estimated using the Adaptive Gaussian Quadrature maximum likelihood estimator (AGQ), with *p* < 0.05 considered statistically significant. Model fitness was appraised using the deviance information criterion (DIC). All analyses used Stata 15.1 (College Station, TX, USA).

The results of random effects were summarised as the VPC and the PCV [31]. The VPC estimates the likelihood of a child developing ARI, which is attributed to the neighbourhood-level variables. A large VPC value implies a high clustering of the possibility of developing ARI in the neighbourhood, and a low VPC shows a homogeneous likelihood of developing ARI. PCV evaluates the proportional difference in the neighbourhood-level variance among the models (Models 0 to 2). Finally, the deviance information criterion (DIC) was used to appraise the model’s fitness.

The first analysis consisted of a multilevel linear regression model of acute respiratory infections. This fitted model provided values for individual and neighbourhood variances. These values were used to calculate the VPC as a percentage of the total individual variance in acute respiratory infection attributed to the neighbourhood level, as shown in Equation (1) [32].

## 3. Results

### 3.1. Descriptive Statistics

The individual and neighbourhood characteristics of the study sample, according to the child’s ARI status, are shown in Table 1. Most of the children lived in rural areas with their mothers (2146, 74.5%) and had mothers who gave birth at home (1320, 77.8%), mothers without access to any of the three mass media (television, radio, and magazines), and married mothers (1,232, 72.3%), and 1272 (76.3%) mothers were manual workers. Around half (1704, 48.8%) of the respondents belonged to the age group 25–34 years and had attained at least primary education (1870, 64.9%) (Table 1). Half of the children were male (1463, 50.8%), and (438, 22.7%) were less than 11 months old. Meanwhile, 1762 (61.1%) were born to mothers with more than four children.

### 3.2. Results

Table 2 presents the log odds of a child having ARI associated with various environmental, neighbourhood, and household covariates, including HAP. The base model showed significant variability in the log odds of developing ARI across the neighbourhoods (τ = 1.30, *p* = 0.001). Based on the VPC estimated by the intercept variance component, 25% of the variability in the log odds of a child developing an ARI was due to neighbourhood-level factors. Next, Model 1 was adjusted for the following explanatory variables: the child’s age, sex, parity, mother’s occupational status and educational attainment, breastfeeding status, type of cooking fuel used in the household, family access to mass media, maternal age, place of delivery, and household wealth status. This adjustment shows that the log odds of a child suffering from ARI were 44% lower for children aged 11–22 months and 41% lower for children aged 22–32 months, compared with the reference category (0–11 months). Children born to mothers aged 15–24 years and those born to mothers aged 25–34 years had 175% and 593% higher log odds of developing ARI, respectively, compared to children of mothers aged 35 years and above, the reference category.

Compared to children whose mothers cook with clean fuel, those who cook with polluting fuel had 215% significantly higher log odds of developing acute respiratory infections. In contrast, the log odds of developing ARI were 44% lower for female children compared to their male counterparts. Similarly, compared to children from the poorest households, children from the wealthiest households had significantly lower log odds of developing acute respiratory infection, and children from households in the fourth quintile (richer) of the wealth index had 58% lower log odds of developing acute respiratory infection than their counterparts from the poorest households.

In addition, compared to children whose mothers were uneducated, children from households with mothers with secondary and higher education had almost 33% less log odds of developing acute respiratory infections. Likewise, compared to children born to mothers without any livelihood, children born to mothers who are professionals or work in white-collar jobs had 20% lower log odds of developing acute respiratory infection.

This adjustment indicated that there is still significant variability in log odds of developing ARI (τ = 1.14, *p* = 0.001), with a slight decrease in community-level variance as compared to the empty model. As reported by VPC, 23.5% of the variability in log odds of developing ARI across communities and neighbourhoods was explained by various individual children and household-level compositional characteristics at the neighbourhood level.

The fully adjusted model, Model 2 in Table 2, determines their effects on the log odds of a child developing an acute respiratory infection due to several explanatory variables, including HAP. As can be seen from Table 2, the log odds of a child developing ARI due to the mothers living in rural areas are increased by 185% as compared to those living in urban areas. Independent of other factors, children of mothers living in the least deprived neighbourhoods had 44% lower log odds of developing an acute respiratory infection than those of the reference category. As shown by the community-level variance, compared to Model 1, the community-level variability in log odds of developing ARI remains significant (τ = 1.12, *p* = 0.001), with 20% of the variance being attributed to both individual- and community-level variables in Model 2. Thus, after controlling for various covariates at both individual and neighbourhood levels, young maternal age, neighbourhood-level socioeconomic disadvantage—a marker of deprivation—and the use of polluting oil for cooking in the household, along with the place of residence, remain statistically significant.

Model 0 was the base model with no exposure variables. Model 1 was adjusted for the child’s characteristics (age, sex, parity) and mother and household characteristics (education, occupation, and wealth index). Model 2 was sequentially adjusted for neighbourhood socioeconomic disadvantage and place of residence, in addition to all the included variables in Model 1.

## 4. Discussion

Using nationally representative data from Chad, this study assessed the effect of individual child- and maternal-level characteristics, including household air pollution from cooking activities, place of residence, and residency in socioeconomic neighbourhoods, on a crucial health outcome: respiratory infection among children under five in the Chad Republic. The results of this study show that neighbourhood-level socioeconomic disadvantage, markers of deprivation, the use of polluting oil for cooking in the household, young maternal age, and place of residence were significant drivers of acute respiratory infections among under-five children in Chad, based on the country′s 2014–2015 CDHS. To the best of the authors′ knowledge, this article is the first nationally representative, population-based study to document the joint effect of socioeconomic development in neighbourhoods and exposure to household air pollution on the likelihood of a child developing ARI in the Republic of Chad, using the multilevel methodology.

The results showed that children living in the least socioeconomically disadvantaged neighbourhoods were 44% less likely than their peers residing in most socioeconomically disadvantaged communities to develop ARI due to several factors, including exposure to polluting cooking fuels. The analysis showed that children with ARI were more likely to be residents of most socioeconomically disadvantaged neighbourhoods, were likely to have mothers who were not working, were rural dwellers, and belonged to the lowest 20% of the wealth quintile.

However, the effect of household wealth status, which had been demonstrated in previous studies [8,14,33,34,35,36,37,38,39,40,41] to be an essential factor in ARI incidence among children, was also confirmed in this study, showing that children from the wealthiest households have a lower likelihood of suffering from ARI as compared to their peers from the poorest families. Similarly, a Bangladeshi study found an association between upper respiratory infections and poverty [1,37]. Poverty may predispose families to domestic pollution caused by using inappropriate cooking fuels [35,42,43,44]. Indeed, wealth is often associated with providing the mother with a good understanding of caring for the home environment [12,34,43]. This finding contrasts with those reported by the ESCALA study in Latin America [45], where there were no differences in the incidence of ARI based on household wealth status.

Individual measures of maternal socioeconomic characteristics, such as education and occupation, have been shown to increase the uptake of preventive child-survival health promotion strategies, including exclusive breastfeeding [12,44,46,47]. More specifically, analysis from this research showed that, with all other factors controlled, children born to mothers with secondary or higher education were less likely to suffer from ARI than their peers whose mothers were not educated at all.

Breastfeeding has previously been shown to offer significant protection against infectious diseases and may be associated with lowering the risk of disease related to HAP, especially in the neonatal and infancy periods [4,7,20,43,48,49]. Earlier studies have found an association between maternal breastfeeding practice and reduced incidence of acute respiratory infection [20,39,45,49,50,51,52,53,54]. However, this study showed no significant relationship between the two variables. This could be attributed to the effect of other variables that may serve as confounders of ARI predisposition.

As noted from the analysis, the incidence of ARI decreased with increasing age among children; this finding is not new but aligns with those of a previous study [55]. The plausible reason for this could be that younger children, due to their age, are more likely to be more attached to their mothers, as some may still be breastfeeding and, therefore, are likely to be more exposed to HAP from cooking fuel.

In this study, the type of fuel used for cooking in households was also significantly associated with ARI. Children from households that used high-polluting fuels had higher odds of ARI than those from households using clean fuels for cooking. This finding is not new and has been reported in many studies [14,20,35,36,44,51,54,56].

Households that use polluting fuels are typically at the lower end of the energy ranking. Polluting fuels are generally made from wood, straw, animal dung, and charcoal. Therefore, it is not surprising that most people using polluting fuels are residing in rural areas, where most are peasant farmers and are poor [54,57]. Additionally, it is sometimes possible for a household to switch from one form of fuel to another, such as from clean fuel to a polluting one, especially when facing financial difficulties. Studies conducted in Bangladesh indicated that households reporting gas as their primary fuel frequently changed to cooking with biomass fuels during shortages of gas supply, which may result in higher concentrations of HAP and weaken associations between HAP and ARI incidence [5,34].

Access to mass media, such as radio or television, has been shown to aid in better assimilation of preventive health through the advertisement of health promotion initiatives [58,59]. Additionally, the fact that preventive health information is often disseminated through social marketing strategies via television, newspapers, and radio stations may have lent support to the association observed in this study. Therefore, this may be why the findings show that children whose mothers had access to all three forms of mass media are less likely to develop ARI than those whose mothers are not in the right direction. Evidence from previous research conducted in several low-income countries has documented this [7,16,23,42,53,60,61].

Unlike what has been reported elsewhere [48], this study found an association between gender differences in the likelihood of developing ARI among children. The result shows that female children were less likely to develop ARI compared to their male counterparts. Hence, the findings were in the same direction as reported among children in India [23,50].

Also, the result of the present research indicates that children born to younger mothers are more likely to develop ARI compared to children whose mothers are much older. This finding is expected and echoes what has been reported elsewhere [6,14,54]. This could be due to older mothers′ caregiving experience and a better understanding of how to protect their children from predisposing factors of ARI. However, a lack of knowledge and education about childcare may contribute to the observed pattern among young mothers.

Furthermore, the results show a wide variation in the likelihood of developing ARI based on a woman′s number of live births. Specifically, the results show that, despite the reported higher birth rate in Chad, which is higher than the regional average, children born to women with at least four live births were less likely to develop ARI than children born to mothers with between one and three live births. The observed finding may be due to the fact that older children might have developed immunity over time compared to their younger siblings. This finding contradicts previous reports in other settings [6,12,62].

In summary, the results corroborate the evidence that polluting biomass fuels, alongside other factors such as household wealth, place of residence, mothers′ educational status, neighbourhood economic development level, and access to educational information through mass media, are associated with an increased likelihood of ARI risk in children in Chad. Therefore, policy-oriented interventions are needed to specifically help reduce women′s use of polluting fuels alongside targeted educational interventions about the deleterious health effects of HAP from cooking fuel in Chad.

### 4.1. Policy Implications

Four major intervention categories have been highlighted to mitigate the impact of indoor air pollution on child ARI: cleaner-burning fuels, improved cookstoves, housing design, and behavioural change. Efforts are required to implement these interventions to achieve the desired result.

### 4.2. Study Strengths and Limitations

The findings of this study have several limitations that should be noted when interpreting the results. The research used secondary data based on a nationally representative survey. However, there are a few potential sources of bias that may be attributed to the selection of participants, misclassification of cooking fuel, and recall bias on the part of the respondents. First, the classification of cooking fuel may be a source of misclassification bias, as some households use a combination of polluting and clean fuels [12,35]. The incidence of ARI/symptoms is self-reported by the mothers, who are prone to recall bias. However, evidence has shown that recall can be accurate depending on the duration of events and what they entail [62].

## 5. Conclusions

The results revealed that household wealth status, HAP through the use of highly polluting cooking fuel, maternal age, never being breastfed, lack of education, residing in rural areas, and living in most socioeconomically disadvantaged communities are significant risk factors for ARI among Chadian children. This study yields numerous significant findings, which, with targeted interventions, can help reduce the ARI burden among children in Chad. The evidence that HAP from cooking fuel, alongside socioeconomic status at individual and neighbourhood levels, and other variables amenable to change, such as maternal education, serve as risk factors for ARI and highlight the need for empowerment initiatives in various forms. Although resources are limited in low-income countries where several health and developmental priorities are competing, tackling each of the drivers of ARI among children is possible. First, exposure to HAP can be prevented with clean cooking fuels and technology. However, this can only be achieved by improving households’ socioeconomic status. For example, the government can provide those who are poor with a means of livelihood through a poverty alleviation programme, helping them escape poverty and enabling them to live in a well-ventilated environment. Being economically able would provide them with money to purchase clean cooking fuel. Second, educational awareness and sensitisation using local languages to spread information about the deleterious effects of HAP is another thing that can be used to bridge the lack of education, which is another risk factor for ARI risk.

## Figures and Tables

**Table 1:** Sample characteristics of children, aged 0–59 months, by ARI status based on CDHS 2014–2015.

		ARI	
Variables	Yes	No	
	*N* (%)	*N* (%)	Total *N* (%)
Child’s age (months)			
0–10	284 (24.8)	154 (19.6)	438 (22.7)
11–21	205 (17.9)	176 (22.4)	381 (19.7)
22–32	203(17.7)	167 (21.3)	370 (19.2)
33+	454 (39.6)	288 (36.7)	742 (38.4)
Child’s sex			
Male	866 (50.8)	597 (50.7)	1463 (50.8)
Female	838 (49.2)	581 (49.3)	1419 (49.2)
Mother’s age (years)			
15–24	503 (29.5)	384 (32.6)	887 (30.8)
25–34	843 (49.5)	562 (47.7)	1704 (48.8)
35+	358 (21.0)	232 (19.7)	590 (20.4)
Mother’s education			
No education	8 (0.5)	9 (0.8)	17 (0.6)
Primary	1119 (65.6)	751 (63.7)	1870 (64.9)
Secondary and higher	577 (33.9)	418 (35.5)	995 (34.5)
Place of delivery			
Home	1320 (77.8)	871 (74.1)	2191 (76.3)
Hospital	377 (22.2)	305 (25.9)	682 (23.7)
Media access (radio, television, and magazine)			
None	1232 (72.3)	793 (67.3)	2024 (70.2)
1	258 (15.1)	189 (16.0)	447 (15.5)
2	50 (2.9)	50 (4.2)	100 (3.5)
3	164 (9.6)	147 (12.5)	311 (10.8)
Mother’s occupation			
Not working	92 (5.5)	77 (6.7)	169 (6.0)
Manual	1272 (76.3)	854 (74.6)	2126 (75.6)
Professional	304 (18.2)	214 (18.7)	517 (18.4)
Wealth index			
Poorest	373 (21.9)	225 (19.1)	598 (20.7)
Poorer	329 (19.3)	226 (19.2)	555 (19.3)
Middle	375 22.0)	239 (20.2)	614 (21.3)
Richer	322 (18.9)	215 (18.3)	537 (18.6)
Richest	305 (17.9)	273 (23.2)	578 (20.1)
Types of cooking fuel			
Polluting	1670 (98.0)	1136 (96.4)	2806 (97.4)
Clean	34 (2.0)	42 (3.6)	76 (2.6)
Parity			
1–3	645 (37.9)	475 (40.3)	1120 (38.9)
4+	1059 (62.1)	703 (59.7)	1762 (61.1)
Maternal breast-feeding status Never breastfed Ever breastfed Still breastfeeding	75 (4.4) 896 (52.3) 742 (43.3)	53 (4.5) 640 (54.8) 476 (40.7)	128 (4.4) 1536 (53.3) 1218 (42.3)
Place of residence Rural Urban	1293 (75.9) 411 (24.1)	853 (72.4) 325 (27.6)	2146 (74.5) 736 (25.5)
Neighbourhood-economic disadvantage Least disadvantaged Most disadvantage	770 (45.2) 934 (54.8)	572 (48.6) 606 (51.4)	1342 (46.6) 1540 (53.4)

**Table 2:** Multilevel logistic regression modelling of factors associated with ARI among Chadian children, CDHS 2014–2015.

Variables	Model 0	Model 1	Model 2
	OR (95% CI)	OR (95% CI)	OR (95% CI)
(*Individual Characteristics*)			
*Types of cooking fuel* Clean fuel Polluting fuel		1 (reference) 3.15 [2.41–4.13] ***	1 (reference) 3.10 [2.39–4.10] ***
*Parity* 1–3 4+		1 (reference) 0.10 [0.17–0.66] *	1 (reference) 0.10 [0.02–0.65] *
Highest level of education No education Primary Secondary and above		1 (reference) 3.02 [0.73–4.60] 0.67 [0.54–0.82] ***	1 (reference) 3.35 [0.72–5.62] 0.66 [0.72–5.62] ***
*Media Access* *None* *1* *2* *3*		1 (reference) 0.66 [0.17–2.60] 0.91 [1.01–1.51] ** 0.18 [0.10–0.54] **	1 (reference) 0.67 [0.17–2.60] 0.66 [0.17–2.60] ** 0.16 [0.17–0.60] **
*Household wealth index* Poorest Poorer Middle Richer Richest		1 (reference) 0.78 [0.42–1.43] 0.69 [0.36–1.34] 0.42 [0.19–0.88] ** 0.34 [0.14–0.83] **	1 (reference) 0.76 [0.41–1.40] 0.54 [0.52–1.11] 0.40 [0.17–0.90] ** 0.34 [0.14–0.89] **
*Occupation* Not working Manual Professional		1 (reference) 1.14 [0.44–2.79] 0.80 [0.65–0.90] *	1 (reference) 0.80 [0.44–2.79] 0.79 [0.63–0.91] *
*Sex of child* Male Female		1 (reference) 0.56 [0.36–0.89] **	1 (reference) 0.54 [0.40–0.89] **
*Child′s age in months* 0–10 11–21 22–32 33+		1 (reference) 0.56 [0.39–0.79] *** 0.59 [0.42–0.85] ** 0.75 [0.56–1.20]	1 (reference) 0.54 [0.39–0.80] *** 0.57 [0.49–0.80] ** 0.80 [0.57–1.31]
Mother’s age(years) 35+ 25–34 15–24		1 (reference) 6.93 [10.52–12.40] *** 2.75 [1.48–5.15] ***	1 (reference) 5.10 [3.88- 12.42] ** 2.78 [3.85- 5.20] **
*Breastfeeding status* Not currently breastfeeding Never breastfed Still breastfeeding		1 (reference) 3.50 [0.52–8.56] 0.48 [0.07–3.08]	1 (reference) 2.96 [0.46–10.00] 0.68 [0.46–1.00]
*Place of birth Delivery* Home Hospital		1 (reference) 0.90 [0.69–1.16]	1 (reference) 0.89 [0.65–1.10]
*Community-level variables* *Place of residence* Urban Rural			1 2.85 [1.73–4.75] ***
*Neighbourhood Economic Disadvantaged Index* Most disadvantaged Least disadvantaged			1 0.56 [0.44–0.70] ***
*Intercept*	0.15 [0.16–0.20] ***	1.14 [0.03] ***	1.12 [0.03] ***
*Neighbourhood-level variance (SE)*	1.30 [0.04] ***	1.14 [0.03] ***	1.12 [0.03] ***
*VPC (%)*	25	23.5	20.0
*Explained variation PCV (%)* *Model fit statistics*	Reference	12.3	17.5
DIC (−2log likelihood)	3651.40	3012.20	2601.10

OR, odds ratio; CI, confidence interval; SE, standard error; DIC, deviance information criterion; VPC, variance partition coefficient; PCV, proportional change in variance. Successively smaller values of deviance information criterion (DIC) with each subsequent model in the bottom of Table 2 show that each model represents a significant improvement over the previous model and indicates the goodness-of-fit of the model used in this analysis. * *p* < 0.05; ** *p* < 0.001; *** *p* < 0.00.

## Data Availability

Relevant guidelines and regulations carried out all procedures, and data were sourced from this link: https://dhsprogram.com/data/available-datasets.cfm (accessed on 11 November 2024).
